# Integrated Analysis of Circadian and Sleep Signatures in Depression and Schizophrenia Using Multi-Day Actigraphy

**DOI:** 10.3390/bioengineering13040383

**Published:** 2026-03-26

**Authors:** Rama Krishna Thelagathoti, Ka-Chun Siu, Hesham H. Ali, Rohan M. Fernando

**Affiliations:** 1 Molecular Diagnostic Research Laboratory, Boys Town National Research Hospital, Omaha, NE 68010, USA; m.rohan.fernando@boystown.org; 2 College of Allied Health Professions, University of Nebraska Medical Center, Omaha, NE 68198, USA; kcsiu@unmc.edu; 3 College of Information Science & Technology, University of Nebraska Omaha, Omaha, NE 68182, USA; hali@unomaha.edu

**Keywords:** circadian rhythms, actigraphy, sleep, schizophrenia, depression, digital biomarkers, wearable sensors

## Abstract

Sleep abnormalities and circadian rhythm disruptions are frequently observed in psychiatric disorders such as depression and schizophrenia. However, most previous studies have examined circadian rhythms and sleep separately, limiting understanding of how these processes interact within individuals. This study examined circadian and sleep characteristics in depression and schizophrenia compared with healthy controls using multi-day wrist actigraphy. Circadian rhythms were assessed using parametric and non-parametric measures of rest–activity patterns, and sleep metrics were derived using a validated actigraphy-based algorithm. Distinct patterns were observed across diagnostic groups. Schizophrenia showed widespread disruption in daily activity patterns, with altered timing and reduced rhythm strength. Sleep was longer but highly fragmented, with frequent awakenings despite increased time in bed. In contrast, depression showed more limited changes, mainly in activity timing and overall activity levels, while sleep and daily patterns remained closer to controls. A key finding was the identification of distinct circadian–sleep profiles for each condition, with global disruption in schizophrenia and more selective alterations in depression. These findings show that combining circadian and sleep measures provides a clearer understanding of psychiatric disorders and may support monitoring and targeted interventions based on daily behavioral rhythms.

## 1. Introduction

Circadian rhythms are endogenous, near-24 h oscillations that regulate a wide array of physiological processes, including hormone secretion, metabolism, mood regulation, and the sleep–wake cycle [1,2,3]. These rhythms are orchestrated by the suprachiasmatic nucleus (SCN), which synchronizes internal biological timing to environmental cues such as light and social activity [4]. Sleep, one of the most overt manifestations of circadian rhythmicity, plays a crucial role in cognitive functioning, emotional regulation, metabolic homeostasis, and overall health [5,6,7]. Disruptions in either circadian timing or sleep continuity can impair neurocognitive performance, increase psychiatric vulnerability, and contribute to long-term cardiometabolic risk [8,9]. Given the close link between circadian rhythms and sleep, evaluating them together is important for understanding behavioral and clinical outcomes.

Circadian abnormalities and sleep disturbances are highly prevalent in psychiatric disorders, especially major depressive disorder (MDD) and schizophrenia. Depression is frequently associated with delayed sleep timing, reduced circadian amplitude, irregular rest–activity patterns, and sleep maintenance difficulties [10,11,12]. Changes in REM sleep, increased awakenings, and delayed circadian phase are commonly reported features, reflecting dysregulation of the circadian–homeostatic interaction [13]. Schizophrenia, in contrast, is characterized by more pronounced circadian disruption, including blunted 24 h amplitude, loss of day–night differentiation, reduced entrainment to zeitgebers, and highly fragmented behavioral rhythms [14,15,16].

Actigraphy has become a central tool for objectively quantifying 24 h rest–activity patterns and sleep in psychiatric populations, particularly major depressive disorder (MDD) and schizophrenia [17,18]. A recent meta-analysis [19] showed that depressed participants exhibit shorter and more fragmented sleep, lower sleep efficiency, and dampened circadian rest–activity rhythms compared with healthy controls, including reduced relative amplitude and greater intradaily variability of activity. In another study by Difrancesco et al. [20] used 14-day actigraphy to relate depressive symptom dimensions to physical activity, sleep, and circadian metrics. They found that lower daytime activity and reduced relative amplitude were consistently associated with more severe depressive symptoms across mood/cognition, somatic/vegetative, and sleep domains. Kang et al. [21] demonstrated that dampened circadian rhythmicity and delayed timing of sleep and activity were robustly associated with current and remitted MDD, suggesting that circadian blunting may reflect a trait marker.

In schizophrenia and related psychotic disorders, multiple actigraphy-based meta-analyses and empirical studies have characterized profound disruption of both sleep continuity and circadian rhythmicity. Meyer et al. [22] reported longer time in bed and total sleep time, longer sleep latency, greater wake after sleep onset, reduced motor activity, and altered circadian amplitude and stability in schizophrenia patients compared with controls. Mayeli et al. [23], studying schizophrenia spectrum disorders (SSD), compared residential and outpatient SSD patients with healthy controls over seven days of actigraphy, showing shared and distinct abnormalities in sleep duration, M10, intradaily variability, interdaily stability, and other RAR metrics, which were further linked to negative symptom severity [24]. Rest–activity rhythm metrics have also been connected to physical health burden in schizophrenia. Mahmood et al. [17] used community-based actigraphy to compare people living with schizophrenia to non-psychiatric controls and showed that RAR disruption (e.g., lower interdaily stability, higher intradaily variability, blunted amplitude) was associated with adverse metabolic indicators, including hyperglycemia and insulin resistance, suggesting that circadian fragmentation may be a pathway linking psychosis to cardiometabolic risk.

Existing studies have typically examined either parametric circadian measures or non-parametric rest–activity rhythm metrics, but few studies have combined both approaches in the same analysis. Similarly, sleep in psychiatric populations is often assessed using self-report or polysomnography, which may not capture long-term, real-world patterns [25]. To our knowledge, few studies have jointly evaluated parametric circadian measures, non-parametric rhythm metrics, and actigraphy-derived sleep measures across control, depression, and schizophrenia groups within the same dataset. The primary aim of this study is to characterize circadian rhythms and sleep patterns across these groups using wrist actigraphy. This study focuses on describing group differences rather than developing diagnostic models.

The present study addresses these gaps by combining different circadian and sleep measures derived from actigraphy. We integrate parametric circadian measures, non-parametric metrics, and actigraphy-based sleep measures to describe patterns across diagnostic groups. This approach allows us to examine daily activity rhythms, sleep timing, and sleep continuity together. Based on the prior literature, we hypothesize that (1) individuals with schizophrenia and depression will show differences in circadian rhythms compared to controls; (2) schizophrenia will show greater disruption in circadian timing and more fragmented sleep than depression; and (3) circadian timing, particularly acrophase, will vary across age groups, while other circadian measures and sleep continuity will show fewer differences. The main outcomes include circadian measures from actigraphy, such as mesor, amplitude, and acrophase, along with non-parametric metrics including interdaily stability, intradaily variability, relative amplitude, L5, and M10. Sleep outcomes include total time in bed, total sleep time, longest sleep duration, wake after sleep onset, number of awakenings, and sleep efficiency. Together, these measures allow a combined assessment of circadian rhythms and sleep across groups.

## 2. Materials and Methods

### 2.1. Dataset Description

The dataset used in this study was constructed by merging two publicly available datasets published by the same research group. The first dataset, Depresjon, contains wrist actigraphy data from 55 adults: 23 individuals diagnosed with unipolar or bipolar depressive disorder and 32 healthy control participants [26]. The second dataset, Psykose, includes 22 individuals diagnosed with schizophrenia as well as the same 32 healthy controls [27]. Because the healthy participants were the same across both datasets, the two sources were merged to form a unified dataset comprising three mutually exclusive groups: (1) depressive disorder, (2) schizophrenia, and (3) healthy controls. In all analyses, individuals with depressive or schizophrenic disorders were considered the condition groups, while the healthy participants served as the control group.

All participants were monitored using a body-worn wearable sensor embedded in an Actigraph Actiwatch (Model: Actiwatch, Cambridge Neurotechnology Ltd., Cambridge, UK), a widely used and validated device for measuring rest–activity behavior in clinical and research settings [28]. The Actiwatch contains a piezoelectric accelerometer that records movement intensity as activity counts, where higher counts reflect greater motor activity. The devices sampled movement data at 32 Hz and captured accelerations above 0.05 g per minute. Participants wore the device continuously on their right wrist during routine daily activities without imposed behavioral constraints, allowing for naturalistic monitoring of rest–activity patterns over multiple days. The datasets analyzed in this study are publicly available, and detailed information regarding recruitment procedures, diagnostic assessments, and clinical characterization is provided in the original source publications.

Each participant originally contributed up to 13 days of motor activity data, although some provided fewer days. To ensure sufficient temporal coverage for reliable circadian and sleep estimation, we included only participants with at least eight days of valid actigraphy data. To examine potential age-related differences, participants were categorized into three age groups based on established demographic ranges: Young Adults (24–34 years), Middle-aged Adults (35–49 years), and Over 50 Adults (50–69 years). The distribution across these conditions and groups is shown in Table 1. And demographic characteristics are presented in Table 2.

### 2.2. Metrics

All raw actigraphy data were collected in 1 min epochs and preprocessed following standard procedures recommended for long-term ambulatory monitoring. Preprocessing steps included timestamp alignment, removal of non-wear periods based on sustained inactivity thresholds, normalization of activity counts, and merging of multi-day files into continuous time series. To facilitate circadian and sleep analysis, each participant’s data were segmented into 24 h windows aligned to the local clock time, ensuring consistent day–night boundaries. From these preprocessed time series, we computed two major classes of behavioral measures: (1) circadian rhythm features, including parametric (cosinor-based) and non-parametric rest–activity rhythm metrics, which jointly characterize rhythmic strength, amplitude, stability, and fragmentation; (2) sleep metrics, derived using a validated algorithm to classify 1-min epochs as sleep–wake classification algorithm, enabling estimation of sleep quantity, sleep efficiency, and night-to-night continuity.

These measures were selected because both circadian and sleep metrics are grounded in the strong bidirectional relationship between 24 h rhythmicity and nocturnal sleep regulation. Circadian rhythm features quantify stability, amplitude, timing, and fragmentation of daily behavioral cycles, which are known to differ across psychiatric conditions such as depression and schizophrenia [3,10,16]. Sleep metrics quantify sleep quantity, efficiency, and continuity, parameters widely disrupted in these disorders and strongly linked to functional outcomes [2,19]. Integrating these measures provides a comprehensive behavioral phenotype that captures both global circadian organization and nighttime sleep dynamics, enabling a multidimensional comparison of rhythm–sleep signatures across diagnostic groups.

### 2.3. Circadian Rhythm Metrics

Circadian rhythm characteristics were assessed using two complementary analytic frameworks: parametric cosinor modeling and non-parametric rest–activity rhythm (NPCRA) metrics. Parametric circadian rhythms were estimated using 24 h single-component cosinor analysis, which models behavioral activity as a sinusoidal function and yields three key parameters: mesor (rhythm-adjusted mean activity), amplitude (half the peak-to-trough oscillation), and acrophase (time of peak activity). These measures quantify the strength and timing of 24 h behavioral rhythms and are widely applied in chronobiological research involving both healthy and psychiatric populations [1,4,10,14].

To complement the parametric analysis, we derived non-parametric metrics based on the rest–activity rhythm framework, which is robust to irregularities and does not assume sinusoidal structure [1,5,6]. These included: Interdaily Stability (IS), reflecting the consistency of the rhythm across days; Intradaily Variability (IV), reflecting rhythm fragmentation and frequency of transitions between rest and activity; Relative Amplitude (RA), capturing the contrast between the most active 10 h (M10) and the least active 5 h (L5); and the L5/M10 metrics themselves, which quantify nighttime rest quality and daytime activity intensity, respectively. These measures index circadian robustness, fragmentation, and entrainment, and have been shown to differentiate depression and schizophrenia from healthy controls in previous work [6,8,14,15,16,17].

### 2.4. Sleep Metrics

Sleep–wake behavior was quantified using the Cole–Kripke algorithm, a validated and widely used actigraphy-based sleep scoring method [29]. Each 1-min epoch was classified as sleep or wake based on weighted activity counts, enabling the computation of standardized sleep metrics. For each participant, nightly sleep profiles were summarized into: Total Time in Bed (TIB), Total Sleep Time (TST), Sleep Efficiency (SE = TST/TIB), Wake After Sleep Onset (WASO), Number of Awakenings, and Longest Uninterrupted Sleep Duration [30]. Sleep timing features, including sleep onset and final wake time, were extracted from the temporal structure of sleep epochs.

These sleep features jointly capture both sleep quantity (TIB, TST) and sleep quality (WASO, awakenings, SE), as well as continuity and restorative potential (longest sleep duration). Prior research has demonstrated that depression is associated with delayed timing and maintenance insomnia [11,12,13], whereas schizophrenia is characterized by severe fragmentation and decreased sleep efficiency [15,16,17,18]. Thus, these sleep metrics provide clinically relevant indicators of nocturnal disruption and serve as a critical complement to circadian rhythm measures in understanding psychiatric conditions.

### 2.5. Statistical Analysis

All statistical analyses were performed using Python (version 3.12.6) and standard scientific libraries. Prior to analysis, all circadian and sleep metrics were averaged within each participant across the available multi-day recordings to obtain stable individual-level estimates. Descriptive statistics (mean ± standard deviation) were computed for all variables within each diagnostic group (Control, Depression, Schizophrenia) and each age category (Young Adults, Middle-aged Adults, Over 50).

Because actigraphy-derived measures typically exhibit non-normal distributions and can contain skewed or heavy-tailed values, non-parametric statistical tests were used for all between-group comparisons. Group differences in circadian metrics (parametric and non-parametric) and sleep metrics were evaluated using pairwise Mann–Whitney U tests, with contrasts computed for:

(1) Depression vs. Control;
(2) Depression vs. Schizophrenia;
(3) Control vs. Schizophrenia.

For age-stratified analyses, the following contrasts were evaluated:

(1) Young Adults vs. Middle-aged Adults;
(2) Young Adults vs. Over 50 Adults;
(3) Middle-aged Adults vs. Over 50 Adults.

To control multiple comparisons within each set of analyses, Bonferroni correction was applied to the resulting *p*-values. Adjusted *p*-values are reported in the corresponding results tables, and statistical significance was determined using the Bonferroni-corrected threshold (*p* < 0.05). Effect sizes for pairwise Mann–Whitney U comparisons were calculated using rank-biserial correlation. Approximate post hoc statistical power for the primary comparisons was estimated based on the observed effect sizes and group sample sizes to evaluate the sensitivity of the analyses.

## 3. Results

This section presents the findings from the circadian and sleep analyses conducted on the merged actigraphy dataset. Circadian rhythm outcomes are reported using both parametric cosinor metrics (mesor, amplitude, acrophase) and non-parametric rest–activity rhythm measures (IS, IV, RA, L5, M10), derived through the statistical procedures described in the Methods. Group-level comparisons for each circadian metric were evaluated using non-parametric statistical tests, and results are summarized in detailed tables including mean values, standard deviations, and pairwise *p*-values. To complement the hypothesis-testing framework, effect sizes were calculated where appropriate to quantify the magnitude of group differences. Statistical significance was set at *p* < 0.05, and all *p*-values were reported without correction unless otherwise indicated. For transparency, full results were tabulated, including mean ± SD for each metric across groups and the corresponding *p*-values for each pairwise comparison. In addition to circadian findings, sleep metrics such as total sleep time, efficiency, WASO, and number of awakenings were also presented in tables.

### 3.1. Circadian Rhythm

**Figure 1:**
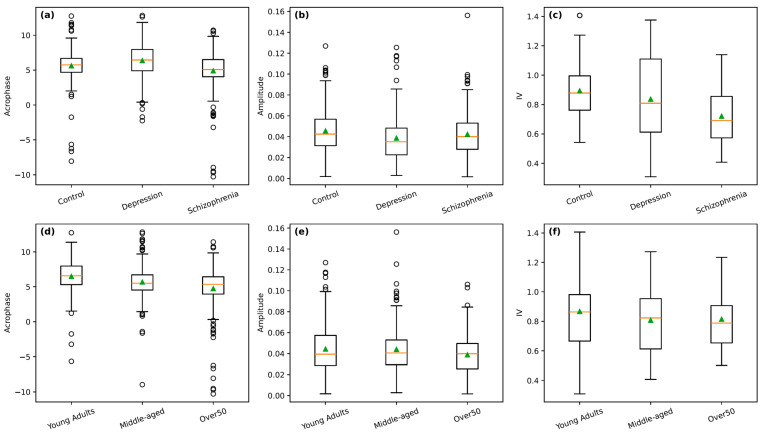
Circadian rhythm metrics across diagnostic and age groups. Box plots illustrate subject-level distributions of selected circadian rhythm metrics, including acrophase, amplitude, and intradaily variability (IV), computed from multi-day actigraphy recordings. The top row (panels (**a**–**c**)) depicts differences across diagnostic groups, and the bottom row (panels (**d**–**f**)) depicts differences across age groups. Acrophase is expressed in hours, amplitude in normalized activity units, and IV is a dimensionless measure of rhythm fragmentation. Boxes indicate the interquartile range with medians (orange line), whiskers show the range excluding outliers, and mean values are indicated by overlaid markers (green arrow).

Group differences in circadian rhythmicity were evident across both parametric and non-parametric measures (shown in Table 3 and Table 4, distributional characteristics of key circadian rhythm measures in Figure 1). Among the parametric metrics, Controls displayed significantly higher mesor and amplitude than both Depression and Schizophrenia, indicating stronger overall 24 h rhythmicity (Bonferroni-adjusted *p* < 0.001 for Control vs. Depression; Bonferroni-adjusted *p* < 0.001 for Control vs. Schizophrenia). Acrophase timing also differed, with Schizophrenia showing significantly earlier peak activity relative to both Depression and Controls (Bonferroni-adjusted *p* < 0.001 and Bonferroni-adjusted *p* < 0.001, respectively), while Depression exhibited a delayed acrophase compared to Controls (*p* = 0.0004). These findings suggest that schizophrenia is associated with phase-advanced and dampened rhythms, whereas depression shows reduced rhythm strength and delayed timing.

Non-parametric circadian measures showed similar directional patterns but did not remain statistically significant after Bonferroni correction. Interdaily Stability (IS) and Relative Amplitude (RA) were lowest in Schizophrenia, consistent with weaker day-to-day regularity and diminished day–night contrast, although group differences were not statistically significant after correction. Intradaily Variability (IV) was significantly lower in Schizophrenia than in Controls; however, this difference did not remain statistically significant following Bonferroni correction. suggesting a smoother, less fragmented activity profile in schizophrenia—likely reflecting overall reduced activity levels rather than improved rhythm consolidation. M10 values were lower in Depression than in Controls but this difference was also not statistically significant after Bonferroni adjustment. Taken together, these results indicate that rhythm amplitude, mean activity, and timing differ most markedly in schizophrenia, with depression showing intermediate disturbances and controls displaying the strongest rhythmic profiles.

Age-stratified analyses showed more subtle patterns. Parametric metrics revealed no significant differences in mesor or amplitude across age groups, indicating overall stability of rhythm strength and mean activity from young adulthood through older age. However, acrophase differed significantly across all comparisons, with Young Adults exhibiting the latest peak activity, followed by Middle-aged, and Over 50 showing the earliest acrophase (Bonferroni-adjusted *p* < 0.001 for Young vs. Mid and Young vs. Over 50; Bonferroni-adjusted *p* = 0.03 for Mid vs. Over 50). This progressive shift toward earlier peak activity suggests an age-related advance in circadian phase, consistent with known chronobiological aging effects.

Non-parametric circadian metrics exhibited modest, non-significant age effects. IS and RA increased slightly with age, suggesting marginally stronger day-night structure among older adults, while IV decreased slightly from younger to older age groups, reflecting marginal reductions in rhythm fragmentation. L5 and M10 also did not differ significantly, indicating comparable nighttime rest intensity and daytime motor activity across age groups. Overall, age influenced circadian timing more strongly than rhythm strength or regularity, with acrophase advancing noticeably across age categories. Post hoc power analyses indicated that the primary circadian comparisons involving mesor, amplitude, and acrophase generally demonstrated moderate to high statistical power, particularly for contrasts between the schizophrenia and control groups, where effect sizes were larger. In contrast, several comparisons involving depression and some non-parametric rhythm metrics showed smaller effect sizes and correspondingly lower statistical power, suggesting that non-significant findings for these metrics should be interpreted cautiously.

The distributional characteristics of key circadian rhythm measures are illustrated in Figure 1, which presents box plots of acrophase, amplitude, and intradaily variability stratified by diagnostic group (panels a–c) and age group (panels d–f). Consistent with the statistical results, the box plots show that individuals with schizophrenia exhibit earlier acrophase timing and reduced rhythm amplitude compared with both controls and individuals with depression, alongside a generally lower median IV, indicating altered temporal organization and dampened rhythmicity (Figure 1a–c). Depression displays intermediate distributions, with delayed acrophase relative to controls but substantial overlap in amplitude and IV. Age-stratified box plots reveal a clear progressive advance in acrophase from young adults to older adults (Figure 1d), while amplitude and IV show overlapping distributions across age groups with no marked differences (Figure 1e,f). Overall, the box plots visually reinforce the tabulated findings by highlighting pronounced shifts in circadian timing and rhythm strength in schizophrenia and age-related phase advances, while underscoring the relative stability of non-parametric rhythm regularity across age groups.

### 3.2. Sleep

Across diagnostic groups, individuals with schizophrenia showed the most marked alterations in sleep (as shown in Table 5). They spent significantly more time in bed (566 ± 83 min) than both controls (444 ± 72 min) and individuals with depression (540 ± 90 min). Total sleep time followed a similar pattern, with schizophrenia showing higher values (470 ± 91 min) than controls (393 ± 57 min), while differences with depression (431 ± 86 min) were not statistically significant (*p* = 0.06). Notably, individuals with schizophrenia had more awakenings (1.44 ± 0.86) than controls (0.77 ± 0.51), indicating greater sleep fragmentation. Also, WASO was higher in both depression (109 ± 64 min) and schizophrenia (85 ± 49 min) compared with controls (51 ± 35 min), although these differences were not statistically significant. In contrast, the control group exhibited higher sleep efficiency than schizophrenia and depression (90% in controls, 81% in depression, 84% in schizophrenia). Nevertheless, depression showed values closer to controls across most measures, with no significant differences in time in bed, total sleep time, or fragmentation indices. Overall, schizophrenia was characterized by longer but more disrupted sleep, whereas depression showed milder deviations.

Sleep metrics showed limited variation across age groups (Table 6). Total time in bed increased modestly from young adults (475 ± 72 min) to middle-aged (517 ± 107 min) and older adults (527 ± 98 min), but these differences were not statistically significant (all *p* ≥ 0.98). Total sleep time followed a similar pattern, increasing from 416 ± 61 min in young adults to 441 ± 83 min in older adults, again without significant differences (*p* ≥ 0.45). Measures of sleep continuity, including wake after sleep onset (59 ± 36 min in young adults vs. 86 ± 59 min in older adults) and number of awakenings (0.85 ± 0.53 vs. 1.33 ± 0.89), were slightly higher with age but did not reach statistical significance (all *p* = 1.00). Sleep efficiency remained stable across groups (89% in young adults vs. 85% in older groups). Effect sizes were small, indicating minimal age-related differences. Overall, age had little impact on sleep quantity or fragmentation in this sample, with broadly consistent sleep patterns across the lifespan.

**Figure 2:**
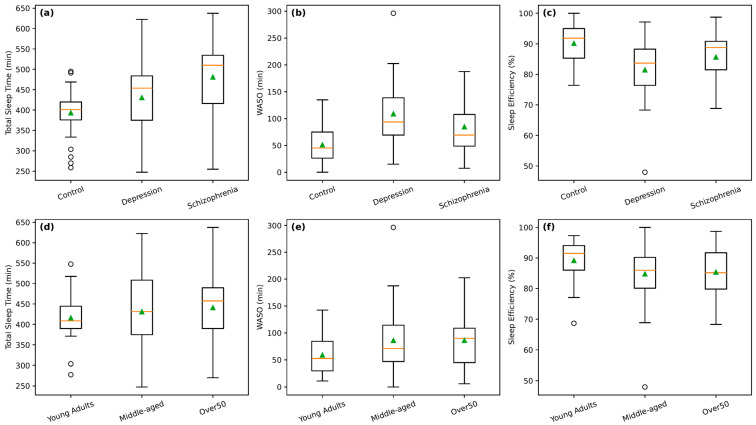
Sleep metrics across diagnostic and age groups. Box plots show the distribution of key sleep metrics derived from wrist actigraphy, including total sleep time, WASO, and sleep efficiency. The top row (panels (**a**–**c**)) presents comparisons across diagnostic groups (control, depression, and schizophrenia), while the bottom row (panels (**d**–**f**)) shows the same metrics stratified by age group (young adults, middle-aged adults, and adults over 50 years). Boxes represent the interquartile range with the median indicated by the central line (orange line); whiskers denote the data range excluding outliers, and mean values are shown by overlaid markers (green arrow).

The distributional patterns of key sleep metrics are further illustrated in Figure 2, which presents box plots of total sleep time, WASO, and sleep efficiency stratified by diagnostic group (panels a–c) and age group (panels d–f). Consistent with the tabulated results, the box plots show that individuals with schizophrenia exhibit markedly higher total sleep time and WASO compared with both controls and individuals with depression, alongside greater inter-individual variability, indicating prolonged but fragmented sleep (Figure 2a,b). This pattern suggests that although individuals with schizophrenia spent longer periods in bed and obtained greater estimated sleep time, their sleep was more fragmented—with higher WASO and more awakenings—leading to lower overall sleep efficiency compared with healthy controls. Although median sleep efficiency appears modestly higher in schizophrenia and depression than in controls, substantial overlap across groups is evident, reflecting the non-significant pairwise differences observed in statistical testing (Figure 2c). Age-stratified box plots similarly demonstrate longer sleep duration and increased WASO in the Over 50 group relative to younger adults, while sleep efficiency remains broadly comparable across age categories (Figure 2d–f). Overall, these visualizations corroborate the statistical findings by highlighting the pronounced variability and fragmentation of sleep in schizophrenia and the more modest, quantity-driven age-related differences in sleep.

Taken together, the results reveal distinct patterns of sleep behavior across clinical conditions and age groups. Schizophrenia is characterized by markedly fragmented yet prolonged sleep, depression shows largely preserved sleep compared with controls, and healthy individuals exhibit the most consolidated sleep. Age-related effects are more modest and primarily reflect differences in sleep duration rather than fragmentation.

## 4. Discussion

In this study, we examined circadian rhythms and sleep patterns across diagnostic and age groups using actigraphy. Circadian parameters showed meaningful differences across diagnostic groups, particularly in mesor, amplitude, and acrophase, indicating changes in overall activity level, rhythm strength, and timing of peak activity. These findings suggest that circadian rhythms are altered rather than absent, with shifts in timing and reduced rhythm robustness in clinical groups. This pattern is consistent with previous research showing reduced activity levels, dampened rhythms, and phase shifts in schizophrenia and depression [14,31]. In addition, acrophase differed significantly across age groups, indicating age-related shifts in the timing of daily activity. In contrast, non-parametric measures of rhythm stability (IS, IV, RA) remained similar across groups, suggesting that the overall structure of daily rhythms was preserved. Alongside these circadian findings, sleep metrics showed clearer clinical differences. Individuals with schizophrenia spent more time in bed and slept longer, but their sleep was more disrupted, with more awakenings and greater nighttime activity. Depression showed patterns similar to controls. Overall, these results may indicate that circadian timing changes and sleep fragmentation together characterize sleep–wake disturbances, with fragmentation being the most prominent feature in schizophrenia.

The pattern of longer but more fragmented sleep in schizophrenia is consistent with previous research. Several studies have shown that individuals with schizophrenia often experience increased sleep duration but reduced sleep efficiency and more awakenings [14,32,33]. This apparent contradiction reflects poor sleep quality despite extended time in bed. Sleep fragmentation has also been linked to cognitive impairment, symptom severity, and reduced daytime functioning in psychiatric populations [34,35]. Our findings support this view and suggest that fragmented sleep may be a more important marker in addition to total sleep time. In contrast, depression showed less consistent differences, which aligns with prior work indicating that sleep in depression is more variable and heterogeneous [36,37].

Medication effects likely contribute to both the sleep and circadian findings observed in this study. Antipsychotic medications are known to increase total sleep time and reduce activity levels, which may explain the higher mesor and longer sleep duration seen in the schizophrenia group [38,39]. At the same time, these medications can influence circadian timing by shifting or dampening biological rhythms, potentially contributing to the observed differences in acrophase [40]. Despite increasing sleep duration, antipsychotics do not necessarily improve sleep quality and may be associated with persistent sleep fragmentation and altered sleep architecture, including frequent awakenings. Antidepressants also have mixed effects on sleep and circadian rhythms, with some agents improving sleep continuity while others disrupt REM sleep or shift circadian phase [41,42]. The relatively modest differences between the depression and control groups in our study may reflect these variable effects.

Age-related analyses further clarified the distinction between clinical and demographic influences on circadian and sleep behavior. Age effects were most evident in circadian timing, with older adults showing significantly earlier acrophase compared to younger and middle-aged groups, consistent with well-established age-related phase advances [22,25]. In contrast, mesor, amplitude, and non-parametric measures (IS, IV, RA, L5, M10) did not differ significantly across age groups, indicating that overall rhythm strength and stability were largely preserved with aging. Similarly, sleep metrics, including total sleep time, time in bed, WASO, number of awakenings, and sleep efficiency, did not show statistically significant differences between age groups, although older adults displayed numerically higher sleep duration and time in bed. These findings suggest that age primarily influences the timing (phase) of circadian rhythms rather than their amplitude, regularity, or sleep continuity.

## 5. Limitations and Future Work

This study has certain limitations. Although actigraphy provides a practical and unobtrusive method for long-term monitoring, it remains an indirect measure of sleep and does not capture sleep stages, microarousals, or neurophysiological markers obtainable from polysomnography. The Cole–Kripke scoring algorithm, while widely validated, may exhibit reduced accuracy in clinical populations with abnormal motor activity, such as schizophrenia. The sample size—while balanced across conditions—remains relatively modest, particularly for age-stratified analyses, and may limit the detection of smaller but meaningful group differences. Sleep onset latency could not be reliably estimated due to the absence of recorded bedtime or lights-off markers in the actigraphy dataset. Therefore, sleep onset latency has been excluded from the analysis. Another limitation of this study is that the depression group included both unipolar and bipolar cases, and we did not conduct subgroup analyses to examine potential differences between these diagnostic categories. Similarly, although antipsychotic use was prevalent in the schizophrenia group, detailed analyses of medication type, dose, and duration were beyond the scope of the present work. Because the primary objective was to characterize cross-diagnostic circadian and sleep phenotypes using a unified analytic framework, these clinically relevant factors were not modeled separately. Future studies should examine diagnostic subtypes and medication effects to determine their specific contributions to circadian and sleep alterations.

Another limitation of this study is that age-based analyses were conducted across the combined sample of patients and healthy controls rather than stratified within each diagnostic group. Because the primary objective was to characterize cross-diagnostic circadian and sleep patterns, age effects were explored at the overall sample level. Future studies with larger cohorts should examine age-related circadian and sleep changes separately within each diagnostic category to better isolate disorder-specific aging effects. Future research should seek to validate these findings in larger and more diverse cohorts and benefit from integrating additional physiological signals such as light exposure, temperature rhythms, and melatonin profiles to obtain more precise estimates of circadian phase and amplitude. Longitudinal designs are needed to determine whether circadian and sleep abnormalities act as predictive markers for symptom fluctuation, relapse, or treatment response.

## 6. Conclusions

This study provides a combined assessment of circadian rhythmicity and sleep patterns across individuals with depression, schizophrenia, and healthy controls using parametric, non-parametric, and actigraphy-derived sleep metrics. The findings indicate that schizophrenia is associated with notable alterations in circadian timing and activity level (mesor, amplitude, and acrophase), along with greater sleep fragmentation and longer time in bed, whereas non-parametric measures of rhythm stability showed limited differences across groups. Individuals with depression showed more modest changes, particularly in circadian timing, while most sleep metrics were comparable to those of controls. Age-related effects were primarily observed in circadian phase (acrophase), with minimal differences in other circadian or sleep measures. Overall, these results suggest that circadian timing and sleep continuity may provide complementary information for understanding group differences, although the observed effects were not uniform across all metrics. The findings also support the use of actigraphy as a practical tool for capturing real-world patterns of activity and sleep. While certain measures, such as acrophase, amplitude, and indices of sleep fragmentation (e.g., WASO and awakenings), appeared informative in this dataset, the present analyses are descriptive and do not establish diagnostic markers. Future studies with larger samples and longitudinal designs are needed to determine whether combinations of circadian and sleep measures can reliably characterize disorder-related patterns or support clinical applications.

## Figures and Tables

**Table 1:** Sample composition by diagnostic group and age category.

Category	Subcategory	Description	Count (*n*)
Diagnostic group	Depression	Diagnosed with depression	23
	Schizophrenia	Diagnosed with schizophrenia	22
	Healthy controls	No psychiatric diagnosis	32
Total sample			77
Age category	Young adults	24–34 years	23
	Middle-aged adults	35–49 years	31
	Over 50 adults	50–69 years	23
Total sample			77

**Table 2:** Demographic characteristics.

	Condition Group		Control Group	
Static	Mean	SD	Mean	SD
Days	12.6	2.3	12.6	2.7
Age	42.8	11	38.2	13
MADRS1	22.7	4.8		
BPRS	50	8.5		
Static	Total	Percentage (%)	Total	Percentage (%)
Gender (Male)	13	58%	12	37.5%
Under antipsychotic medication	17	77%		

**Table 3:** Parametric and non-parametric circadian rhythm metrics (mean ± SD) across control, depression, and schizophrenia groups. Pairwise comparisons were performed using Mann–Whitney U tests with Bonferroni-corrected *p*-values. Effect sizes are reported as rank-biserial correlations (r).

Metric	Control (Mean ± SD)	Depression (Mean ± SD)	Schizophrenia (Mean ± SD)	*p* (Dep vs. Ctrl)	*p* (Dep vs. Schz)	*p* (Ctrl vs. Schz)
	Parametric metrics					
Mesor	0.06 ± 0.02	0.04 ± 0.02	0.05 ± 0.02	**<0.001** (r = −0.42)	0.06 (r = −0.14)	**<0.001** (r = 0.33)
Amplitude	0.05 ± 0.02	0.04 ± 0.02	0.04 ± 0.02	**<0.001** (r = −0.23)	**0.04** (r = −0.12)	0.06 (r = 0.11)
Acrophase	5.63 ± 2.43	6.37 ± 2.60	4.90 ± 3.17	**<0.001** (r = 0.20)	**<0.001** (r = 0.32)	**<0.001** (r = 0.16)
	Nonparametric metrics					
IS	0.00 ± 0.00	0.01 ± 0.01	0.00 ± 0.00	1.00 (r = 0.23)	1.00 (r = −0.02)	1.00 (r = −0.24)
IV	0.89 ± 0.22	0.84 ± 0.27	0.72 ± 0.19	1.00 (r = −0.13)	1.00 (r = 0.25)	0.10 (r = 0.43)
RA	0.92 ± 0.07	0.91 ± 0.07	0.89 ± 0.07	1.00 (r = −0.23)	1.00 (r = 0.15)	1.00 (r = 0.28)
L5	0.00 ± 0.00	0.00 ± 0.00	0.00 ± 0.00	1.00 (r = −0.04)	1.00 (r = −0.26)	1.00 (r = −0.25)
M10	0.09 ± 0.03	0.07 ± 0.03	0.08 ± 0.03	0.22 (r = −0.39)	1.00 (r = −0.08)	0.84 (r = −0.31)

Note: Bold *p*-values indicate statistically significant differences (*p* < 0.05).

**Table 4:** Parametric and non-parametric circadian rhythm metrics (mean ± SD) across three age groups. Pairwise comparisons were performed using Mann–Whitney U tests with Bonferroni-corrected *p*-values. Effect sizes are reported as rank-biserial correlations (r).

Metric	Young Adults (Mean ± SD)	Middle-Aged (Mean ± SD)	Over 50 (Mean ± SD)	*p* (Young vs. Mid)	*p* (Young vs. Over 50)	*p* (Mid vs. Over 50)
	Parametric metrics					
Mesor	0.05 ± 0.02	0.05 ± 0.02	0.05 ± 0.02	0.84 (r = −0.01)	0.33 (r = 0.16)	0.34 (r = 0.05)
Amplitude	0.04 ± 0.02	0.04 ± 0.02	0.04 ± 0.02	0.86 (r = −0.01)	0.10 (r = 0.10)	0.05 (r = 0.11)
Acrophase	6.49 ± 2.33	5.69 ± 2.34	4.73 ± 3.35	**<0.001** (r = 0.28)	**<0.001** (r = 0.39)	**0.03** (r = 0.12)
	Nonparametric metrics					
IS	0.002 ± 0.001	0.003 ± 0.002	0.005 ± 0.001	1.00 (r = 0.02)	1.00 (r = 0.10)	0.18 (r = 0.09)
IV	0.87 ± 0.26	0.81 ± 0.24	0.82 ± 0.21	1.00 (r = 0.09)	1.00 (r = 0.10)	0.97 (r = 0.01)
RA	0.92 ± 0.05	0.90 ± 0.08	0.90 ± 0.08	1.00 (r = −0.11)	1.00 (r = −0.12)	0.80 (r = −0.02)
L5	0.00 ± 0.00	0.01 ± 0.00	0.00 ± 0.00	1.00 (r = −0.15)	1.00 (r = −0.15)	0.60 (r = 0.05)
M10	0.09 ± 0.03	0.09 ± 0.03	0.08 ± 0.03	1.00 (r = −0.02)	0.58 (r = −0.08)	0.60 (r = −0.07)

**Table 5:** Sleep metrics (mean ± SD) across control, depression, and schizophrenia groups, with pairwise Mann–Whitney U test *p*-values. Effect sizes are reported as rank-biserial correlations (r).

Metric	Control (Mean ± SD)	Depression (Mean ± SD)	Schizophrenia (Mean ± SD)	*p* (Dep vs. Ctrl)	*p* (Dep vs. Schz)	*p* (Ctrl vs. Schz)
Total Time in Bed	444 ± 72	540 ± 90	566 ± 83	1.00 (r = 0.09)	**0.02** (r = −0.57)	**<0.001** (r = −0.71)
Total Sleep Time	393 ± 57	431 ± 86	470 ± 91	1.00 (r = 0.16)	0.06 (r = −0.53)	**<0.001** (r = −0.72)
Longest Sleep Duration	349 ± 60	338 ± 96	371 ± 97	1.00 (r = 0.16)	0.30 (r = −0.43)	**<0.001** (r = −0.68)
WASO	51 ± 35	109 ± 64	85 ± 49	1.00 (r = −0.02)	0.67 (r = −0.38)	0.50 (r = −0.37)
Number of Awakenings	0.77 ± 0.51	1.53 ± 0.75	1.44 ± 0.86	1.00 (r = 0.04)	0.06 (r = −0.52)	**<0.001** (r = −0.64)
Sleep Efficiency (%)	90	81	84	0.48 (r = 0.13)	1.00 (r = 0.09)	1.00 (r = −0.07)

Note: Bold *p*-values indicate statistically significant differences (*p* < 0.05).

**Table 6:** Sleep metrics (mean ± SD) across three age groups, with pairwise Mann–Whitney U test *p*-values. Effect sizes are reported as rank-biserial correlations (r).

Metric	Young Adults (Mean ± SD)	Middle-Aged (Mean ± SD)	Over 50 (Mean ± SD)	*p* (Young vs. Mid)	*p* (Young vs. Over 50)	*p* (Mid vs. Over 50)
Total Time in Bed	475 ± 72	517 ± 107	527 ± 98	1.00 (r = 0.07)	0.98 (r = 0.35)	1.00 (r = −0.19)
Total Sleep Time	416 ± 61	431 ± 100	441 ± 83	1.00 (r = 0.08)	0.45 (r = 0.40)	1.00 (r = −0.22)
Longest Sleep Duration	361 ± 51	343 ± 96	355 ± 90	1.00 (r = 0.07)	0.56 (r = 0.39)	1.00 (r = −0.25)
WASO	59 ± 36	86 ± 61	86 ± 59	1.00 (r = −0.03)	1.00 (r = 0.23)	1.00 (r = −0.20)
Number of Awakenings	0.85 ± 0.53	1.33 ± 0.78	1.33 ± 0.89	1.00 (r = 0.15)	1.00 (r = −0.01)	1.00 (r = −0.01)
Sleep Efficiency (%)	89	85	85	1.00 (r = 0.07)	1.00 (r = 0.19)	1.00 (r = 0.09)

## Data Availability

Data is available in the public domain for download.

## Abbreviations

The following abbreviations are used in this manuscript:

TST	Total Sleep Time
TIB	Time in Bed
WASO	Wake After Sleep Onset
SE	Sleep Efficiency
LSD	Longest Sleep Duration
IS	Interdaily Stability
IV	Intradaily Variability
RA	Relative Amplitude
L5	Least active 5 h period
M10	Most active 10 h period
Mesor	Midline Estimating Statistic of Rhythm
Amplitude	Half the difference between the peak and trough of the rhythm
Acrophase	Timing of the peak of the circadian rhythm
SD	Standard Deviation
*p*	*p*-value (statistical significance)
r	Rank-biserial correlation (effect size)
Ctrl	Control group
Dep	Depression
Schz	Schizophrenia
